# Seismic anisotropy evidence for dehydration embrittlement triggering intermediate-depth earthquakes

**DOI:** 10.1038/s41598-017-02563-w

**Published:** 2017-06-01

**Authors:** Jian Wang, Dapeng Zhao, Zhenxing Yao

**Affiliations:** 1 Key Laboratory of Earth and Planetary Physics, Institute of Geology and Geophysics, Chinese Academy of Sciences, Beijing, 100029 China; 2 Department of Geophysics, Tohoku University, Sendai, 980-8578 Japan

## Abstract

It has been proposed that dehydration embrittlement of hydrous materials can trigger intermediate-depth earthquakes and form a double seismic zone in a subducting slab. Seismic anisotropy may provide a possible insight into intermediate-depth intraslab seismicity, because anisotropic properties of minerals change with varying water distribution, temperature and pressure. Here we present a high-resolution model of P-wave radial anisotropy tomography of the Japan subduction zone down to ~400 km depth, which is obtained using a large number of arrival-time data of local earthquakes and teleseismic events. Our results reveal a close correlation between the pattern of intermediate-depth seismicity and anisotropic structures. The seismicity occurs in portions of the Pacific and Philippine Sea slabs where positive radial anisotropy (i.e., horizontal velocity being faster than vertical one) dominates due to dehydration, whereas the inferred anhydrous parts of the slabs are found to be aseismic where negative radial anisotropy (i.e., vertical velocity being faster than horizontal one) dominates. Our anisotropic results suggest that intermediate-depth earthquakes in Japan could be triggered by dehydration embrittlement of hydrous minerals in the subducting slabs.

## Introduction

Double seismic zones, where intraslab earthquakes occur in two distinct dipping planes at depths of ~50–~250 km, are observed widely in subduction zones regardless of the oceanic plate age, convergence rate, or stress orientation^1–3^. Proposed hypotheses for the physical mechanism of intermediate-depth (50–250 km) earthquakes include transformational faulting^4^, thermal shear instability^5, 6^, and dehydration embrittlement^7, 8^. Currently, the hypothesis of dehydration embrittlement is considered to be the leading mechanism, which suggests that earthquakes in double seismic zones are linked to brittle failure associated with dehydration reactions of hydrous minerals in the slab crust and uppermost mantle^9–12^. This popular hypothesis has been tested mainly by thermal-petrologic models and laboratory deformation experiments for hydrous minerals in subducting slabs^11, 13^. Although the existence of hydrous minerals in subducting slabs has been demonstrated by studies of seismic tomography^14^ and receiver functions^15^, there still remain questions where and how water migrates in a subduction zone.

Seismic anisotropy in the upper mantle results from crystallographic preferred orientations (CPOs) of minerals (prominently olivine) induced by dislocation creep^16–18^. Laboratory experiments have revealed that the varying types of olivine fabrics (e.g., A-, B-, C-, D-, and E-type), which relate to different olivine slip systems, cause different seismic anisotropy structures due to water content, temperature and stress/pressure^17, 18^. For example, as decribed by Karato *et al*.^17^, A- and D-type olivine fabrics generally exist in the oceanic and continental lithosphere with poor water content; B-type olivine fabrics are induced mainly by increased water content at high stress and cause azimuthal anisotropy normal to mantle flow, which is proposed to explain trench-parallel azimuthal anisotropy in the fore-arc mantle wedge of subduction zones; C-type olivine fabrics are induced by increased water content at lower stress than that of B-type olivine fabrics, and possibly cause negative radial anisotropy (i.e., vertical velocity being faster than horizontal one) in horizontal mantle flow and weak positive radial anisotropy (i.e., horizontal velocity being faster than vertical one) in vertical cylindrical flow; E-type olivine fabrics are induced by moderate water content and observed in island arc environments^19^. Hence, seismic anisotropy may provide important insights into intermediate-depth seismicity because both of them are closely associated with water migration and distribution in subduction zones.

**Figure 1:**
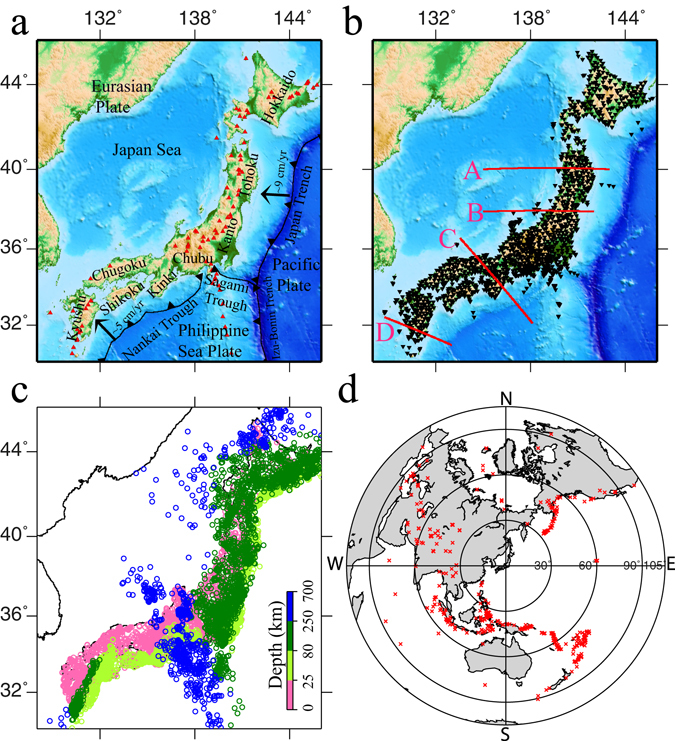
Map of the Japan Islands and surrounding regions. (**a**) Tectonic settings. The red triangles denote active arc volcanoes. (**b**) Distribution of 1656 seismic stations (black triangles) used in this study, which belong to the Japanese national universities, Japan Meteorological Agency (JMA), and the High-sensitivity seismic network (Hi-net). The red lines show locations of the four profiles shown in Fig. 3. (**c**) Epicentral distribution of 7350 local earthquakes (color circles) used in this study. The colors denote the focal depths. (**d**) Epicentral distribution of the 376 teleseismic events (red crosses) used in this study. This Figure is generated using GMT 4.5.3 (http://www.soest.hawaii.edu/gmt/) developed by Wessel and Smith^59^.

The subduction processes of the old Pacific plate and the young Philippine Sea (PHS) plate (Fig. 1) control seismotectonics of the Japan Islands. The Japan subduction zone is an ideal place to study the physical mechanism of intermediate-depth intraslab seismicity, because the earthquake distribution exhibits significant changes during the subduction processes (Supplementary Fig. S1). In the old Pacific slab, intraslab seismicity occurs down to the mantle transition zone^20^. In the young PHS slab, however, the intraslab seismicity occurs down to ~60 km depth and the aseismic PHS slab exists down to ~150 km depth under Chubu, Kinki and Shikoku^20–22^. Beneath Kyushu, the intraslab seismicity occurs down to ~200 km depth, whereas the aseismic PHS slab is revealed down to ~500 km depth^20, 21, 23^.

Previous studies have revealed significant seismic anisotropy in the upper mantle at depths <150 km beneath Japan^24–27^, however, little is known about seismic anisotropy in the deep upper mantle at depths >~200 km. The existence of seismic anisotropy in the deep upper mantle is controversial^16^. The deformed materials in the deep upper mantle are suggested to be isotropic because diffusion creep rather than dislocation creep is dominant there, which may not develop preferred mineral orientation^28^. Although the mechanism causing seismic anisotropy in the deep upper mantle is still in debate, anisotropic structures may exist at least down to the mantle transition zone^29^ and the lower mantle^30^.

We have developed a tomographic method for determining 3-D distribution of P-wave velocity (Vp) anisotropy^27^, in which Vp radial anisotropy is estimated with assumed vertical hexagonal symmetry by adding one anisotropic parameter. In this study, we apply this method to obtain the first model of 3-D Vp radial anisotropy in the upper mantle beneath the entire Japan Islands using a large number of high-quality arrival-time data of local earthquakes and teleseismic events. Our present results shed new light on the relation between seismic anisotropy and water distribution in the upper mantle.

## Results

We used 901,726 P-wave arrival times of 7350 local earthquakes and 39,175 P-wave relative travel-time residuals of 376 teleseismic events recorded by 1656 stations (Fig. 1b–d) during a period of 1998–2012. The relative travel-time residuals of teleseismic events are calculated from waveforms using the multichannel cross-correlation technique^31^. A slightly modified J-B model^32^ with varying depths of the Conrad and Moho discontinuities^33–35^ is adopted as the starting Vp model (Supplementary Fig. S2). A 3-D grid with a grid interval of 0.5° in latitude, 0.4° in longitude and at depths of 8, 20, 40, 60, 80, 120, 160, 200, 240, 280, 320 and 380 km is set up in the modeling space to express the 3-D isotropic Vp variation and Vp radial anisotropy. The maximum ray-azimuthal gap angle^36^ (MRAGA), which denotes the azimuthal coverage of ray paths around a grid node (Supplementary Fig. S3), is set to be 45° during inversions. The optimal values of damping and smoothing parameters for both the isotropic Vp variation and Vp radial anisotropy are obtained by considering the balance between reduction of the root-mean-square travel-time residual and smoothness of the inverted 3-D Vp model (see Supplementary Fig. S4). The local earthquakes are relocated during the inversion process.

**Figure 2:**
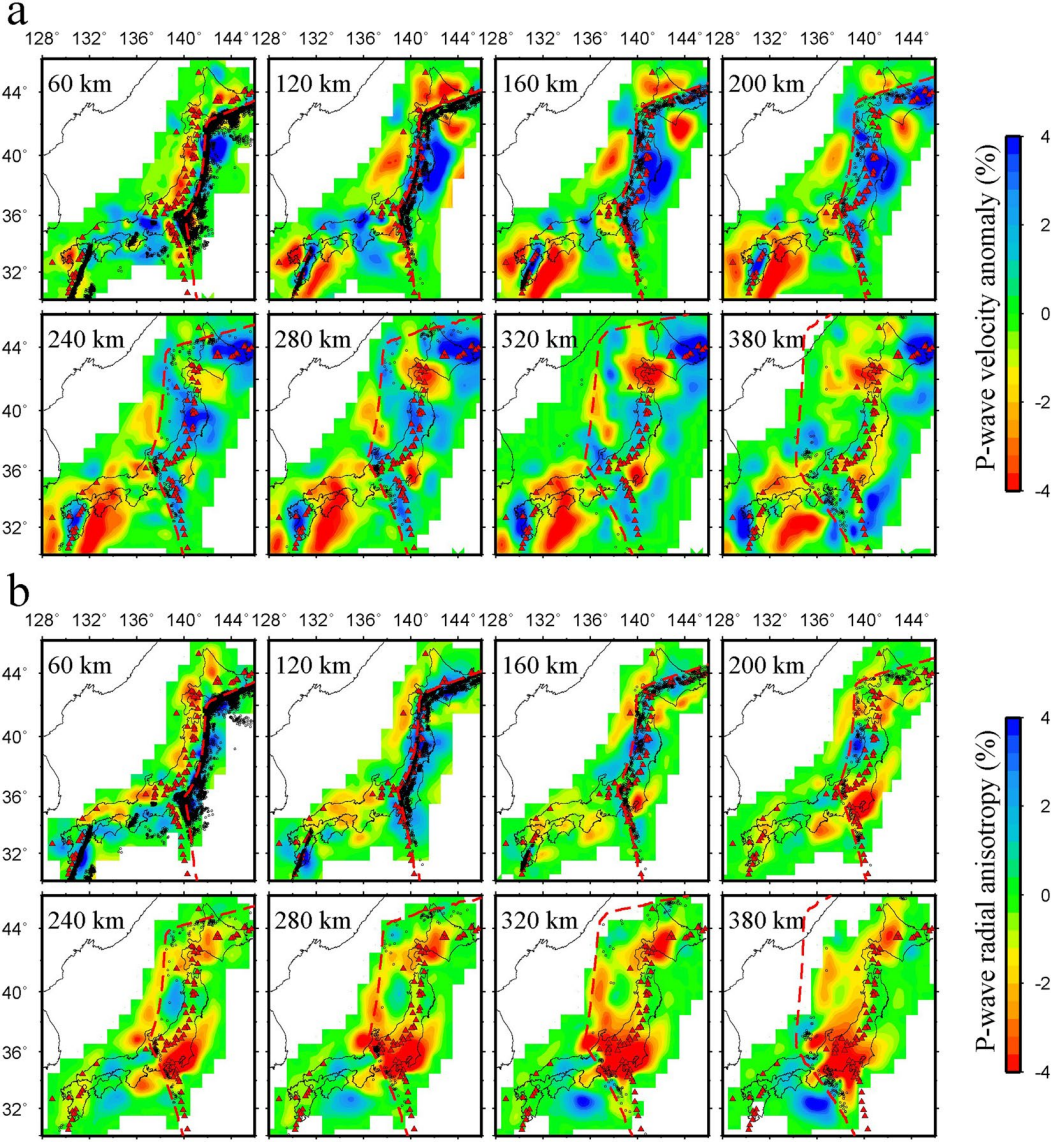
Tomographic images at selected depths beneath Japan. (**a**) Isotropic P-wave velocity images. The red and blue colors denote low and high velocities, respectively. (**b**) Images of P-wave radial anisotropy. The red and blue colors denote negative and positive radial anisotropies, respectively. The scales for the isotropic Vp anomaly and radial anisotropy amplitude are shown on the right. A 3-D grid with a grid interval of 0.5° in latitude, 0.4° in longitude and at depths of 8, 20, 40, 60, 80, 120, 160, 200, 240, 280, 320, and 380 km is set up in the study region for the tomographic inversion. The red dashed line in each map denotes the location of the upper boundary of the subducting Pacific slab at each depth^37, 38^. The red triangles denote active arc volcanoes. The black dots denote the seismicity during a period of 2002–2007 that occurred within a 10 km depth of each layer. This Figure is generated using GMT 4.5.3 (http://www.soest.hawaii.edu/gmt/) developed by Wessel and Smith^59^.

**Figure 3:**
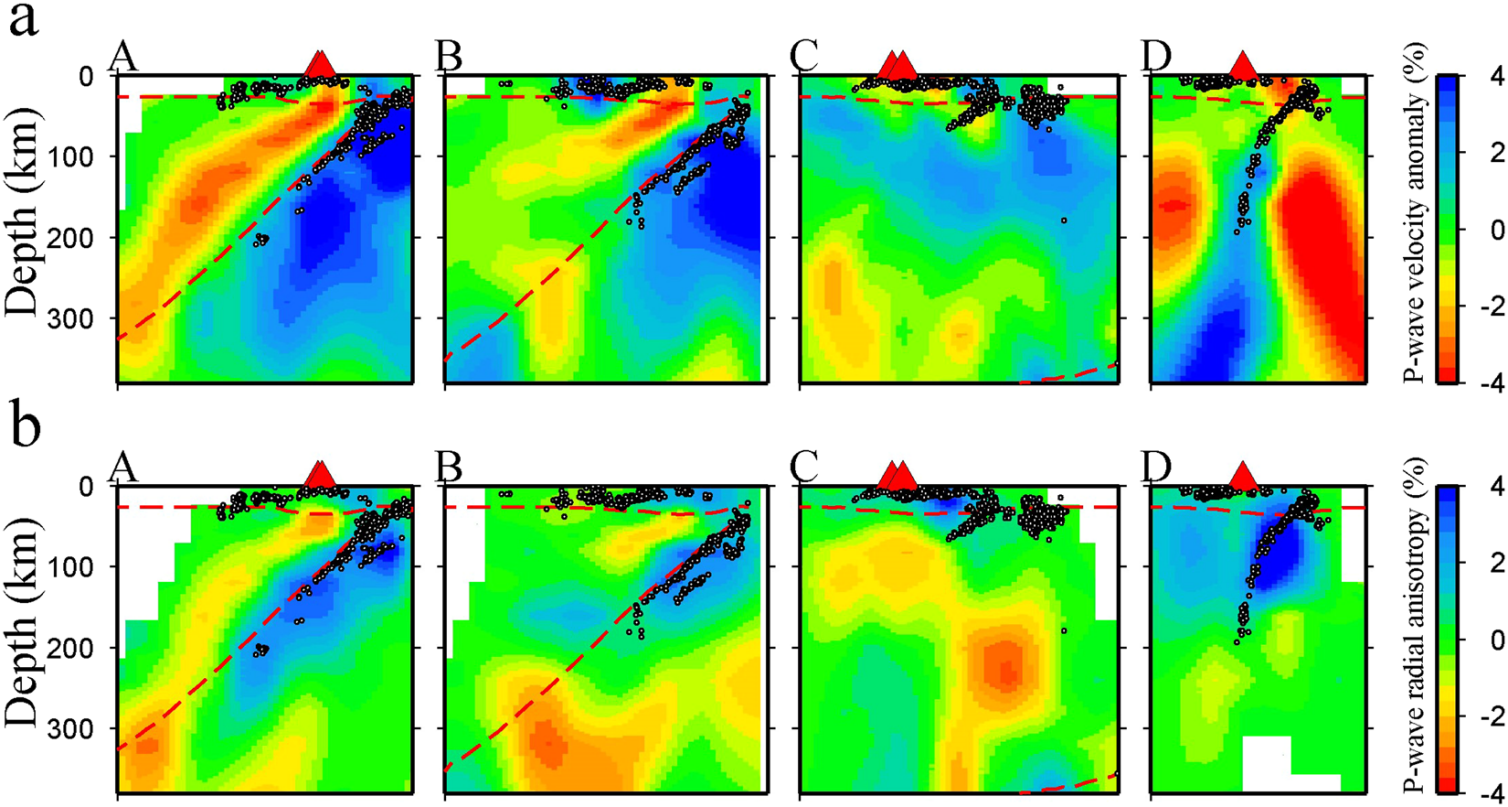
Vertical cross-sections of tomography along the four profiles shown in Fig. 1b. (**a**) Isotropic P-wave velocity images. (**b**) Images of P-wave radial anisotropy. The definitions of the color scales for the isotropic velocity anomaly and radial anisotropy amplitude are the same as those in Fig. 2. The red dashed lines show the Moho discontinuity^33, 35^ and the upper boundary of the subducting Pacific slab^37, 38^, respectively. The red triangles and white dots denote active arc volcanoes and seismicity during a period of 2002–2007 within a 10 km width of each profile. This Figure is generated using GMT 4.5.3 (http://www.soest.hawaii.edu/gmt/) developed by Wessel and Smith^59^.

Figures 2 and 3 show the obtained images of Vp isotropic variation and Vp radial anisotropy at eight selected depths and along four vertical cross-sections, respectively (more images are shown in the Supplementary Figs S5 and S6). The subducting Pacific slab is revealed as a high-velocity (high-V) zone down to the mantle transition zone, and the subducting PHS slab is imaged as a high-V zone with a complex geometry beneath Southwest Japan. Low-velocity (low-V) anomalies appear in the mantle wedge beneath the volcanic front. The estimated upper boundary of the Pacific slab is generally consistent with the previous results denoted as the red dashed lines in Figs 2 and 3, which was estimated from hypocentral locations of intermediate-depth earthquakes^37^ and converted waves at the slab upper boundary^38^.

We conducted a checkerboard resolution test (CRT) and two synthetic tests for the radial anisotropy tomography. In the input model of the CRT, isotropic Vp anomaly and Vp radial anisotropy with an amplitude of ±4% were alternatively assigned to the grid nodes. Random errors in a normal distribution with a standard deviation of 0.15 s were added to the theoretical arrival times. The test results (Supplementary Figs S7 and S8) indicate that the resolution is very good at depths <380 km. In the first synthetic test, the inversion results (Supplementary Figs S5 and S6) were adopted as the input model, and random errors in a normal distribution with a standard deviation of 0.15 s were added to the synthetic arrival times. The second synthetic test is similar to the first one, in the input model the pattern of Vp variations is the same but the anisotropy is opposite. The output results of the synthetic tests (Supplementary Figs S9–S14) indicate that both the isotropic Vp variation and Vp radial anisotropy are well resolved in the upper mantle, suggesting that main features of our tomographic results are reliable.

Compared with our previous study^27^, which shows a good resolution at depths <150 km using only P-wave arrival times of local earthquakes, our present results reveal complex seismic anisotropy structures in the deeper upper mantle, and a close relation between the intraslab seismicity and radial anisotropy. The seismic PHS slab exhibits a positive radial anisotropy (i.e., horizontal Vp being faster than vertical Vp), but the aseismic PHS slab shows a negative radial anisotropy (i.e., vertical Vp being faster than horizontal Vp) beneath Kinki and Kyushu (see Fig. 2 and vertical cross-sections c and d in Fig. 3). The intermediate-depth earthquakes in the Pacific slab occurred mainly in areas of positive radial anisotropy (Fig. 2 and cross-sections a and b in Fig. 3).

## Discussion

Although some small amounts of hydrous minerals (serpentine, antigorite, talc, chlorite, clinoclore, etc.) in the subducting slab are very anisotropic for both P and S waves^39^, we think that seismic anisotropy is induced mainly by olivine CPOs in the upper mantle considering the resolution scale (~40 km × 50 km × 50 km) of our tomographic model. Recent experiments have suggested that the same olivine fabric shows the same seismic anisotropy for S-wave^17^ and P-wave^40^. Since the olivine fabric changes due to different water content, temperature and stress/pressure, we interpret our Vp radial anisotropy results by following and extrapolating the experiment measurements on olivine crystal-fabrics^17, 40^. Similar to the straightforward method by Michibayashi *et al*.^40^, we use the structural framework (x-y-z axes) to denote P-wave velocities in three directions of horizontal flow: V_x_ denotes P-wave velocity in the mantle flow direction (trench-normal direction), V_y_ denotes P-wave velocity perpendicular to the flow direction in the horizontal plane (trench-parallel direction), and V_z_ denotes P-wave velocity in the vertical direction.

**Figure 4:**
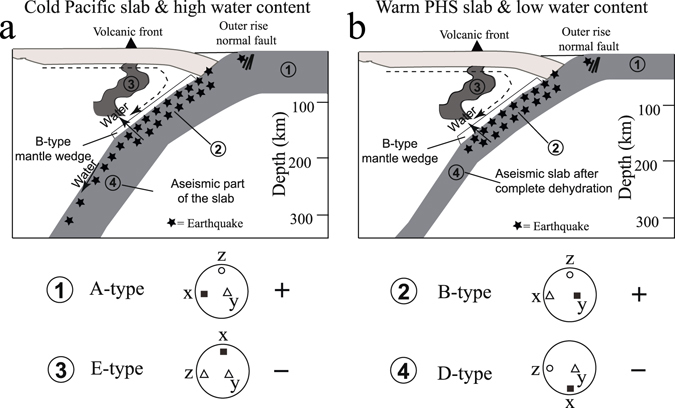
Schematic vertical cross-sections depicting relations between seismic anisotropy, water migration, and double seismic zone in cold (**a**) and warm (**b**) subduction zones. They show the anisotropic structures of the subducting slabs (marked by 1, 2 and 4), the overlying B-type mantle wedge, and the hot mantle upwelling associated with arc volcanism (marked by 3). The dashed arrow denotes the slab-driven corner flow direction. The solid arrow denotes water migration induced by dehydration embrittlement of hydrous materials in the slabs. Possible types of olivine fabrics in different parts are also shown. In each of the big circles below the vertical cross-sections, the solid square, open triangle and open circle denote the directions of the maximum, intermediate and minimum P-wave velocities, respectively. The two open triangles in the circle ③ denote that the axis directions and amounts of the intermediate and minimum P-wave velocities cannot be distinguished by this study. The plus (+) and minus (−) symbols beside the big circles denote positive and negative radial anisotropies, respectively. The velocity features for the types of olivine fabric are derived from the previous studies^17, 40^. This figure is generated by free and open source INKSCAPE 0.91 (https://inkscape.org/en/).

When the oceanic plate is produced (marked by 1 in Fig. 4), its lithospheric mantle is anhydrous because water prefers to dissolve in melting. Hence, the olivine fabric of anhydrous lithospheric mantle is A-type, in which seismic anisotropy is V_x_ > V_y_ > V_z_, that is, the fast Vp direction is trench-normal in azimuthal anisotropy, and positive radial anisotropy occurs. This explanation is consistent with the observation that Pn waves travel faster normal to the mid-ocean ridge^41^.

Large amounts of water entered the oceanic lithospheric mantle from the outer-rise normal faults^42^ and was stored in hydrous minerals when the oceanic plate began to subduct. The dehydration of hydrous minerals in the slab crust and uppermost mantle at intermediate depths may cause brittle failures, which result in double seismic zones within the subducting slab. As shown in Fig. 3, the areas of positive radial anisotropy exist roughly above the high-V zones (the subducting slabs), probably suggesting that the water has been expelled from the subducted slabs into the overlying mantle wedge. The expelled water hydrates the surrounding slab mantle and the overlying mantle wedge, resulting in B-type olivine fabric there (marked by 2 in Fig. 4). Thus the seismic anisotropy is V_y_ > V_x_ > V_z_, that is, trench-parallel azimuthal anisotropy and positive radial anisotropy in the upper part of the subducting slabs and the overlying mantle wedge. This result agrees well with the observed trench–parallel azimuthal anisotropy in the fore-arc mantle wedge and the uppermost parts of the slab mantle beneath Japan by shear-wave splitting measurements^24, 43^ and P-wave anisotropic tomography^27^. However, the conditions inducing the B-type olivine fabrics or serpentinization are still unclear when the water migrates into the overlying mantle wedge, for example, the CPOs of serpentine rather than B-type olivine in a hydrated mantle wedge are proposed to explain a strong trench-parallel anisotropy (1–2 s shear-wave delay time) beneath the Ryukyu arc^26^. Since the anisotropic amplitudes beneath Japan^24, 27, 43^ are smaller than those beneath the Ryukyu arc, the anisotropic features of the hydrated slab mantle (marked by 2 in Fig. 4) and the overlying mantle wedge are induced mainly by B-type olivine fabrics.

The water, migrated from the hydrated overlying mantle wedge^44^, mainly entered melts rather than olivine and induced the mantle upwelling beneath the arc volcanoes. Thus, E-type olivine may be dominant in the mantle upwelling, resulting in negative radial anisotropy, because V_x_ is the maximum P-wave velocity in the vertical flow direction (marked by 3 in Fig. 4). The result is consistent with the observed E-type olivine fabrics of some samples in the island arc environments^19^.

Hydrated materials of the oceanic plate subsequently release fluids induced by the rising temperature of slab during its subduction^45^. The hydrous phases may be completely dehydrated at some critical depth (~200 km), and hydrogen could not be transferred to a greater depth when the slab temperature is relatively high, however, a cold slab may transfer hydrogen even into the lower mantle with staged dehydrations during the subduction process^39^. Because the PHS slab is relatively young (~10–40 m.y.)^46^, it may be anhydrous and could not produce seismicity due to the absence of dehydration embrittlement at its intermediate depths. The olivine fabrics in the anhydrous aseismic PHS slab may change to D-type because of high stress induced by slab bending, for which seismic anisotropy is V_x_ > V_y_ > V_z_. In a number of subduction zones on Earth, the slab geometry generally shows an increasing dip angle at a greater depth in the mantle, possibly affected by trench retreat and slab rollback^47^. Thus, the above-mentioned x and z axes for the olivine fabric in the slab rotated counterclockwise and replaced each other with unchanged y axis, and V_x_ is the velocity in the vertical direction while V_z_ and V_y_ are the velocities in the horizontal plane. As a result, the aseismic PHS slab shows negative radial anisotropy (marked by 4 in Fig. 4b). The same explanation could be applied to the lower part of the cold Pacific slab (marked by 4 in Fig. 4a) at depths of ~240−~320 km where negative radial anisotropy is dominant and induced by D-type olivine fabrics. Our results also show that the intraslab seismicity mainly occurs in areas of positive radial anisotropy (Figs 2 and S5) even down to 380 km depth in the cold Pacific slab. Although it is suggested that the cold Pacific slab can transfer hydrogen into the lower mantle, our results cannot resolve the mechanism of deep earthquakes (>250 km depth) because little is known about the water content and dehydration reaction in the subducting slab in the deeper upper mantle and at the mantle transition zone depth^48^. It is still in question whether the deeper intraslab earthquakes could be generated by dehydration embrittlement, because the water storage capacity for hydrous phases in the cold slab would increase at greater depths^9, 49, 50^. Little correlation is found between seismicity rate and calculated slab dehydration flux at depths >240 km^51^. The hypothesis of transformational faulting on the upper and lower edges of a metastable olivine wedge is applied to explain the seismicity in the deeper double seismic zones observed in Tonga at depths of 350–460 km^52^ and in Izu-Bonin at depths of 300–450 km^53^.

## Conclusions

Seismic anisotropy provides a wealth of new information and direct constraints for understanding geodynamics of the Earth’s interior. We present the first images of 3-D high-resolution P-wave radial anisotropy in the upper mantle beneath the entire Japan Islands. Our results reveal a close relationship between anisotropy and deformation of hydrous minerals and provide new information on the water migration process in the upper mantle of the Japan subduction zone. The present results provide the first direct evidence from seismic anisotropy for dehydration embrittlement of hydrous minerals as the main contributor to generate intermediate-depth earthquakes in subducting slabs.

## Methods

We used P-wave radial anisotropy tomography^27, 54^ to invert for the 3-D anisotropic structure down to a depth of 380 km beneath the Japan Islands. Rocks in the Earth are generally assumed to have the anisotropy with hexagonal symmetry and the P-wave slowness can be written as^55^

1 $$S={S}_{0}(1+{M}_{1}\,\cos (2\theta )),$$

where *S* is the total slowness, *S*_0_ is the average slowness (i.e., isotropic component), *M*_1_ is the parameter for anisotropy, and *θ* is the angle between the propagation vector and the symmetry axis. Thus, for weak radial anisotropy with a vertical hexagonal symmetry axis, equation (1) could be rewritten as^27, 54^

2 $$S={S}_{0}(1+{M}_{1}\,\cos (2i)),$$

where *i* is the incident angle of a ray path. Following equation (2), the total travel time *T* of the ray can be expressed as

3 $$\begin{array}{rcl}T & = & \sum _{k}{T}_{k},\\ {T}_{k} & = & dS=d(1+{M}_{1k}\,\cos (2{i}_{k}))/{V}_{k},\end{array}$$

where *T*_*k*_ is the travel time of the *k*th ray segment with a length *d*, *V*_*k*_ is the isotropic velocity at the middle point of the *k*th ray segment, *M*_1*k*_ is the parameter for the radial anisotropy at the middle point of the *k*th ray segment, and *i*_*k*_ is the incident angle of the *k*th ray segment. As a result, the partial derivatives to the velocity and radial anisotropy are expressed as

4 $$\begin{array}{rcl}\frac{\partial T}{\partial {V}_{k}} & = & -d(1+{M}_{1k}\,\cos (2{i}_{k}))/{V}_{k}^{2},\\ \frac{\partial T}{\partial {M}_{1k}} & = & \frac{d}{{V}_{k}}\,\cos (2{i}_{k}).\end{array}$$

Similar to the isotropic tomography method^56^, the parameters *V*_*k*_ and *M*_1*k*_ are defined at (*φ*, *λ*, *h*), and *f*(*φ*, *λ*, *h*) is calculated by using a linear interpolation function

5 $$f(\varphi ,\lambda ,h)=\sum _{i=1}^{2}\sum _{j=1}^{2}\sum _{k=1}^{2}\,f({\varphi }_{i},{\lambda }_{j},{h}_{k})[(1-|\frac{\varphi -{\varphi }_{i}}{{\varphi }_{2}-{\varphi }_{1}}|)(1-|\frac{\lambda -{\lambda }_{j}}{{\lambda }_{2}-{\lambda }_{1}}|)(1-|\frac{h-{h}_{k}}{{h}_{2}-{h}_{1}}|),$$

where *φ* is latitude, *λ* is longitude, and *h* is the depth from the Earth’s surface, *φ*_*i*_, *λ*_*j*_ and *h*_*k*_ represent the coordinates of the eight grid nodes surrounding the point (*φ*, *λ*, *h*). Thus, a travel-time residual *dT* for the *n*th local earthquake (or a relative travel-time residual *dT* for the *n*th teleseismic event) to the *m*th station can be expressed as

6 $$\begin{array}{rcl}dT & = & {(\frac{\partial T}{\partial \varphi })}_{mn}{\rm{\Delta }}{\varphi }_{n}+{(\frac{\partial T}{\partial \lambda })}_{mn}{\rm{\Delta }}{\lambda }_{n}+{(\frac{\partial T}{\partial h})}_{mn}{\rm{\Delta }}{h}_{n}+{\rm{\Delta }}{T}_{0n}\\  &  & +\sum _{p}(\frac{\partial T}{\partial {V}_{p}}{\rm{\Delta }}{V}_{p})+\sum _{q}(\frac{\partial T}{\partial {M}_{1q}}{\rm{\Delta }}{M}_{1q})+{E}_{mn},\end{array}$$

where *φ*_*n*_, *λ*_*n*_, *h*_*n*_ and *T*_0*n*_ are the latitude, longitude, focal depth and origin time of the *n*th event, respectively; the symbol Δ denotes perturbation of a parameter; *V*_*p*_ is the isotropic velocity at the *p*th node of the 3-D grid net for the isotropic velocity structure; and *M*_1*q*_ is the radial anisotropy parameter at the *q*th node of the 3-D grid net for anisotropy. The grid intervals of the two grid nets can be the same or different. *E*_*mn*_ represents higher-order terms of perturbations and errors in the data. The first four terms on the right side of equation (6) are contributions of the hypocentral parameters, which can be calculated using the method^57^. The equation (6) is solved using the LSQR algorithm^58^ with damping and smoothing regularizations. The amplitude *β* of the radial anisotropy is expressed as

7 $$\beta =\frac{{V}_{ph}-{V}_{pv}}{2{V}_{0}}=\frac{{M}_{1}}{1-{M}_{1}^{2}},$$

where *V*_0_ denotes the average isotropic velocity, *V*_*ph*_ and *V*_*pv*_ are P-wave velocities in the horizontal and vertical directions, respectively. Hence, *β* > 0 denotes that the horizontally propagating P-wave travels faster than the vertical one, while β < 0 denotes that the vertically propagating P-wave travels faster than the horizontal one.

## Electronic supplementary material

Supplementary Information

## References

[1] Brudzinski MR, Thurber CH, Hacker BR, Engdahl ER (2007). Global Prevalence of Double Benioff Zones. Science.

[2] Yamasaki T, Seno T (2003). Double seismic zone and dehydration embrittlement of the subducting slab. J. Geophys. Res..

[3] Shiobara H (2010). Double seismic zone in the North Mariana region revealed by long-term ocean bottom array observation. Geophys. J. Int..

[4] Kao H, Liu LG (1995). A Hypothesis for the Seismogenesis of a Double Seismic Zone. Geophys. J. Int..

[5] Ogawa M (1987). Shear Instability in a Viscoelastic Material as the Cause of Deep-Focus Earthquakes. J. Geophys. Res..

[6] Kelemen PB, Hirth G (2007). A periodic shear-heating mechanism for intermediate-depth earthquakes in the mantle. Nature.

[7] Green HW, Houston H (1995). The Mechanics of Deep Earthquakes. Annu. Rev. Earth Planet. Sci..

[8] Kirby S (1995). Interslab Earthquakes and Phase-Changes in Subducting Lithosphere. Rev. Geophys..

[9] Houston, H. 4.13 - Deep Earthquakes. In: Schubert, G. (ed). *Treatise on Geophysics* (*Second Edition*). Elsevier, Oxford, pp 329–354 (2015).

[10] Peacock SM (2001). Are the lower planes of double seismic zones caused by serpentine dehydration in subducting oceanic mantle?. Geology.

[11] Hacker BR, Peacock SM, Abers GA, Holloway SD (2003). Subduction factory 2. Are intermediate-depth earthquakes in subducting slabs linked to metamorphic dehydration reactions?. J. Geophys. Res..

[12] Brantut N, Passelègue FX, Deldicque D, Rouzaud JN, Schubnel A (2016). Dynamic weakening and amorphization in serpentinite during laboratory earthquakes. Geology.

[13] Jung H, Green HW, Dobrzhinetskaya LF (2004). Intermediate-depth earthquake faulting by dehydration embrittlement with negative volume change. Nature.

[14] Mishra OP, Zhao DP (2004). Seismic evidence for dehydration embrittlement of the subducting Pacific slab. Geophys. Res. Lett..

[15] Kawakatsu H, Watada S (2007). Seismic Evidence for Deep-Water. Science.

[16] Savage MK (1999). Seismic anisotropy and mantle deformation: What have we learned from shear wave splitting?. Rev. Geophys..

[17] Karato S, Jung H, Katayama I, Skemer P (2008). Geodynamic Significance of Seismic Anisotropy of the Upper Mantle: New Insights from Laboratory Studies. Annu. Rev. Earth Planet. Sci..

[18] Mainprice D, Tommasi A, Couvy H, Cordier P, Frost DJ (2005). Pressure sensitivity of olivine slip systems and seismic anisotropy of Earth’s upper mantle. Nature.

[19] Mehl L, Hacker BR, Hirth G, Kelemen PB (2003). Arc-parallel flow within the mantle wedge: Evidence from the accreted Talkeetna arc, south central Alaska. J. Geophys. Res..

[20] Zhao DP, Yanada T, Hasegawa A, Umino N, Wei W (2012). Imaging the subducting slabs and mantle upwelling under the Japan Islands. Geophys. J. Int.

[21] Wang J, Zhao DP (2012). P wave anisotropic tomography of the Nankai subduction zone in Southwest Japan. Geochem. Geophys. Geosyst..

[22] Nakajima J, Hirose F, Hasegawa A (2009). Seismotectonics beneath the Tokyo metropolitan area, Japan: Effect of slab-slab contact and overlap on seismicity. J. Geophys. Res..

[23] Liu X, Zhao DP (2016). P and S wave tomography of Japan subduction zone from joint inversions of local and teleseismic travel times and surface-wave data. Phys. Earth Planet. Inter..

[24] Huang ZC, Zhao DP, Wang LS (2011). Shear wave anisotropy in the crust, mantle wedge, and subducting Pacific slab under northeast Japan. Geochem. Geophys. Geosyst..

[25] Long MD, Silver PG (2008). The Subduction Zone Flow Field from Seismic Anisotropy: A Global View. Science.

[26] Katayama I, Hirauchi H, Michibayashi K, Ando J (2009). Trench-parallel anisotropy produced by serpentine deformation in the hydrated mantle wedge. Nature.

[27] Wang J, Zhao DP (2013). P-wave tomography for 3-D radial and azimuthal anisotropy of Tohoku and Kyushu subduction zones. Geophys. J. Int..

[28] Nicolas, A. Principles of Rock Deformation. Reidel, D., Norwell, Mass, 208 pp (1984).

[29] Yuan KQ, Beghein C (2013). Seismic anisotropy changes across upper mantle phase transitions. Earth Planet. Sci. Lett..

[30] Tsujino N (2016). Mantle dynamics inferred from the crystallographic preferred orientation of bridgmanite. Nature.

[31] Vandecar JC, Crosson RS (1990). Determination of Teleseismic Relative Phase Arrival Times Using Multi-Channel Cross-Correlation and Least-Squares. Bull. Seismol. Soc. Am.

[32] Jeffreys, H. & Bullen, K. Seismological tables. *Abstract Presented at the Annual Meeting of the Seismological Society of Japan* (*2000*), British Association for the Advancement of Science, London, pp 50 (1940).

[33] Zhao DP, Horiuchi S, Hasegawa A (1990). 3-D Seismic Velocity Structure of the Crust and the Uppermost Mantle in the Northeastern Japan Arc. Tectonophysics.

[34] Horiuchi S, Ishii H, Takagi A (1982). Two-Dimensional Depth Structure of the Crust beneath the Tohoku District, the Northeastern Japan Arc. 1. Method and Conrad-Discontinuity. J. Phys. Earth.

[35] Horiuchi S (1982). Two-Dimensional Depth Structure of the Crust beneath the Tohoku District, the Northeastern Japan Arc. 2. Moho Discontinuity and P-Wave Velocity. J. Phys. Earth.

[36] Wang J, Zhao DP (2010). Mapping P-wave anisotropy of the Honshu arc from Japan Trench to the back-arc. J. Asian Earth Sci..

[37] Hasegawa A, Umino N, Takagi A (1978). Double-Planed Deep Seismic Zone and Upper-Mantle Structure in Northeastern Japan Arc. Geophys. J. Roy. Astron. Soc..

[38] Zhao DP, Matsuzawa T, Hasegawa A (1997). Morphology of the subducting slab boundary in the northeastern Japan arc. Phys. Earth Planet. Inter..

[39] Mainprice, D. & Ildefonse, B. Seismic Anisotropy of Subduction Zone Minerals–Contribution of Hydrous Phases. In: Lallemand, S. Funiciello, F. (eds). *Subduction Zone Geodynamics*. Springer Berlin Heidelberg, Berlin, Heidelberg, pp 63–84 (2009).

[40] Michibayashi K (2016). Natural olivine crystal-fabrics in the western Pacific convergence region: A new method to identify fabric type. Earth Planet. Sci. Lett..

[41] Hess H (1964). Seismic anisotropy of the uppermost mantle under oceans. Nature.

[42] Ranero CR, Morgan JP, McIntosh K, Reichert C (2003). Bending-related faulting and mantle serpentinization at the Middle America trench. Nature.

[43] Nakajima J, Hasegawa A (2004). Shear-wave polarization anisotropy and subduction-induced flow in the mantle wedge of northeastern Japan. Earth Planet. Sci. Lett..

[44] Manthilake G, Bolfan-Casanova N, Novella D, Mookherjee M, Andrault D (2016). Dehydration of chlorite explains anomalously high electrical conductivity in the mantle wedges. Sci. Adv..

[45] Ulmer P, Trommsdorff V (1995). Serpentine Stability to Mantle Depths and Subduction-Related Magmatism. Science.

[46] Muller RD, Sdrolias M, Gaina C, Roest WR (2008). Age, spreading rates, and spreading asymmetry of the world’s ocean crust. Geochem. Geophys. Geosyst..

[47] Schellart WP (2010). Evolution of Subduction Zone Curvature and its Dependence on the Trench Velocity and the Slab to Upper Mantle Viscosity Ratio. J. Geophys. Res..

[48] Green HW, Chen WP, Brudzinski MR (2010). Seismic evidence of negligible water carried below 400-km depth in subducting lithosphere. Nature.

[49] Angel RJ, Frost DJ, Ross NL, Hemley R (2001). Stabilities and equations of state of dense hydrous magnesium silicates. Phys. Earth Planet. Inter..

[50] Kohlstedt DL, Keppler H, Rubie DC (1996). Solubility of water in the alpha, beta and gamma phases of (Mg,Fe)(2)SiO4. Contrib. Mineral. Petrol..

[51] Barcheck CG, Wiens DA, van Keken PE, Hacker BR (2012). The relationship of intermediate- and deep-focus seismicity to the hydration and dehydration of subducting slabs. Earth Planet. Sci. Lett..

[52] Wiens DA, Mcguire JJ, Shore PJ (1993). Evidence for Transformational Faulting from a Deep Double Seismic Zone in Tonga. Nature.

[53] Iidaka T, Furukawa Y (1994). Double Seismic Zone for Deep Earthquakes in the Izu-Bonin Subduction Zone. Science.

[54] Wang J, Wu HH, Zhao DP (2014). P wave radial anisotropy tomography of the upper mantle beneath the North China Craton. Geochem. Geophy. Geosyst.

[55] Barclay AH, Toomey DR, Solomon SC (1998). Seismic structure and crustal magmatism at the Mid-Atlantic Ridge, 35 degrees N. J. Geophys. Res..

[56] Zhao DP, Hasegawa A, Horiuchi S (1992). Tomographic Imaging of P-Wave and S-Wave Velocity Structure beneath Northeastern Japan. J. Geophys. Res..

[57] Engdahl ER, Lee WHK (1976). Relocation of Local Earthquakes by Seismic Ray Tracing. J. Geophys. Res..

[58] Paige CC, Saunders MA (1982). Lsqr - an Algorithm for Sparse Linear-Equations and Sparse Least-Squares. Acm. Trans. Math. Software.

[59] Wessel P, Smith WHF (1998). New, improved version of generic mapping tools released. Eos Trans. AGU.

