# Supplementary material for: Seismic anisotropy evidence for dehydration embrittlement triggering intermediate-depth earthquakes
Source: Sci Rep. 2017 Jun 1;7:2613. doi: 10.1038/s41598-017-02563-w (PMC5453959; doi:10.1038/s41598-017-02563-w)
Supplement: Supplementary file 1 — Supplementary Information [file 41598_2017_2563_MOESM1_ESM.pdf]

Supplementary Information for

Seismic anisotropy evidence for dehydration embrittlement triggering intermediate-depth earthquakes

Jian Wang[1,*], Dapeng Zhao[2], Zhenxing Yao[1]

1. Key Laboratory of Earth and Planetary Physics, Institute of Geology and Geophysics, Chinese Academy of Sciences, Beijing 100029, China

2. Department of Geophysics, Tohoku University, Sendai 980-8578, Japan

*Corresponding author. *E-mail address:* jianwang@mail.iggcas.ac.cn

The PDF file includes fourteen figures:

- figure S1.Map showing the isodepth contous of the upper boundary of the subducting Pacific slab and that of the subducting Philippine Sea (PHS) slab.
- figure S2. 1-D P-wave velocity model used in this study.
- figure S3. The definition for the maximum ray-azimuth gap angle (MRAGA).
- figure S4. Trade-off curves for the norm of 3-D velocity model and the RMS travel-time residual for different values of the damping (a) and smoothing (b) parameters.
- figure S5. Map views of isotropic P-wave velocity tomography at different depths.
- figure S6. Map views of P-wave radial anisotropy at different depths.
- figure S7. Results of the checkerboard resolution test for the isotropic Vp tomography at different depths.
- figure S8. Results of the checkerboard resolution test for P-wave radial anisotropy at different depths.
- figure S9. Results of the first synthetic test for the isotropic Vp tomography at different depths.
- figure S10. Results of the first synthetic test for P-wave radial anisotropy at different depths.
- figure S11. Results of the first synthetic test for P-wave velocity and radial anisotropy in vertical cross-sections.
- figure S12. Results of the second synthetic test for the isotropic Vp tomography at different depths.
- figure S13. Results of the second synthetic test for P-wave radial anisotropy at different depths.
- figure S14. Results of the second synthetic test for P-wave velocity and radial anisotropy in vertical cross-sections.

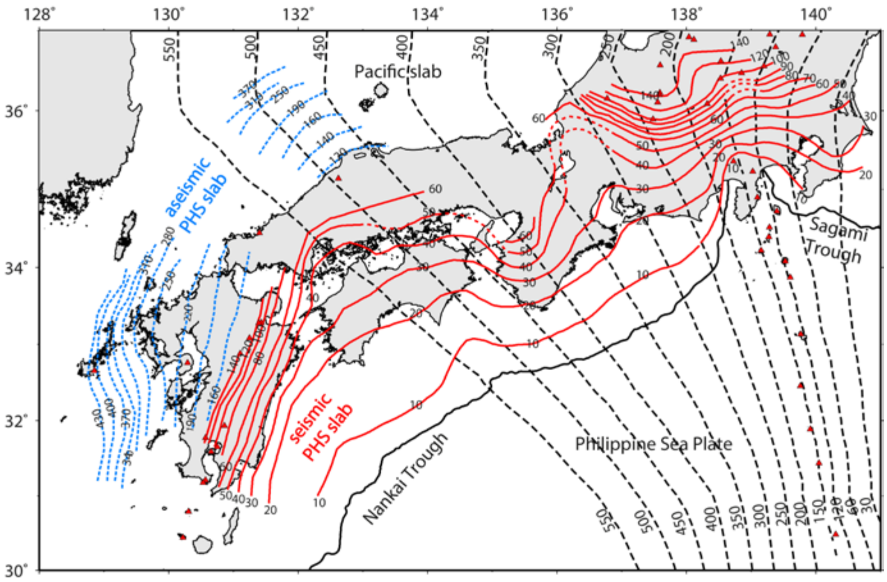

Figure S1. Map showing the isodepth contous of the upper boundary of the subducting Pacific slab and that of the subducting Philippine Sea (PHS) slab. The black dotted lines show the upper boundary of the seismic Pacific slab[37, 38]. The red continuous lines denote the shallow parts of the PHS slab estimated from the seismicity in the PHS slab and local earthquake tomography[22]. The blue dotted lines denote the upper boundary of the aseismic PHS slab estimated from the teleseismic tomography[20]. This Figure is generated using GMT 4.5.3 (http://www.soest.hawaii.edu/gmt/) developed by Wessel and Smith[59].

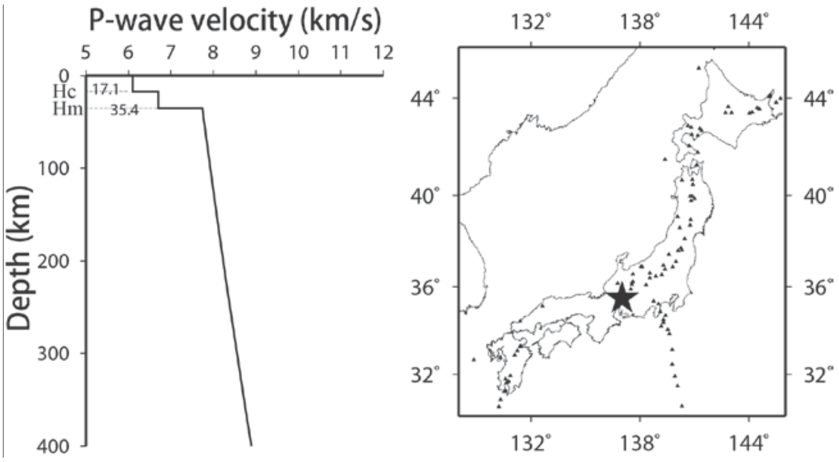

Figure S2. 1-D P-wave velocity model used in this study. Hc and Hm denote the Conrad and Moho depths[33-35], respectively, under the area shown with a star symbol in the map. The black triangles denote the active arc volcanoes. This figure is generated using GMT 4.5.3 (http://www.soest.hawaii.edu/gmt/) developed by Wessel and Smith[59].

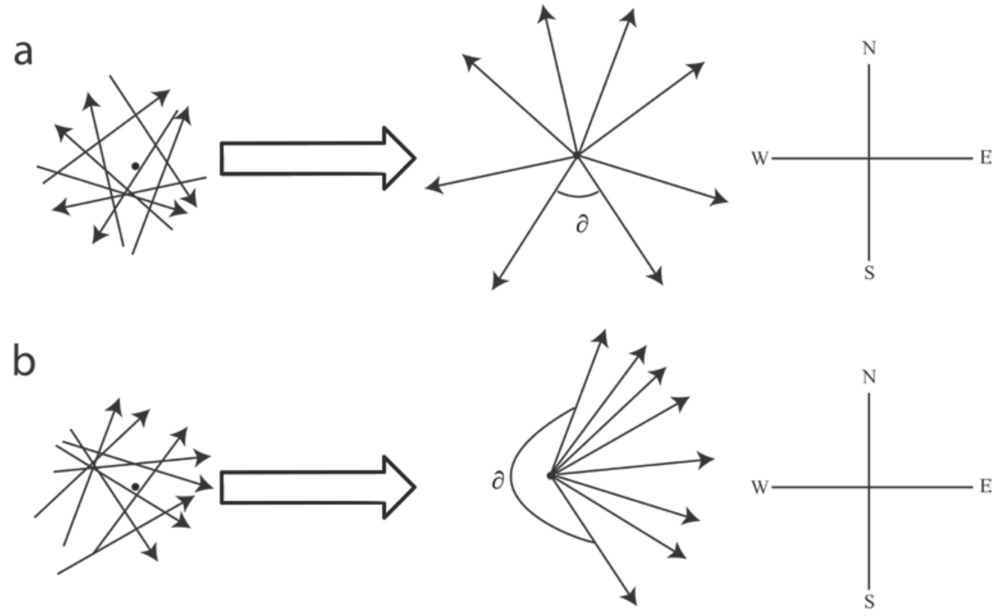

Figure S3. The definition for the maximum ray-azimuth gap angle (MRAGA). ∂ denotes the MRAGA[36]. In the anisotropic tomography, travel-time data are inverted for radial anisotropy at each grid node when the rays pass through the node in all azimuthal directions similar to the pattern as shown in (a) rather than that in (b). In this study, the MRAGA is set to be 45°. This figure is generated by free and open source INKSCAPE 0.91 (https://inkscape.org/en/).

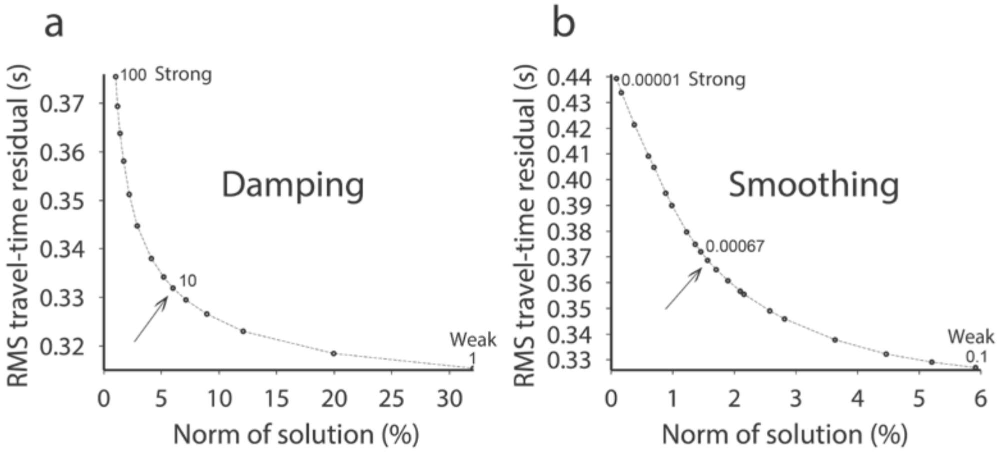

Figure S4. Trade-off curves for the norm of 3-D velocity model and the RMS travel-time residual for different values of the damping (a) and smoothing (b) parameters. The numbers beside the arrows denote the optimal damping and smoothing parameters, respectively. This figure is generated using GMT 4.5.3 (http://www.soest.hawaii.edu/gmt/) developed by Wessel and Smith[59].

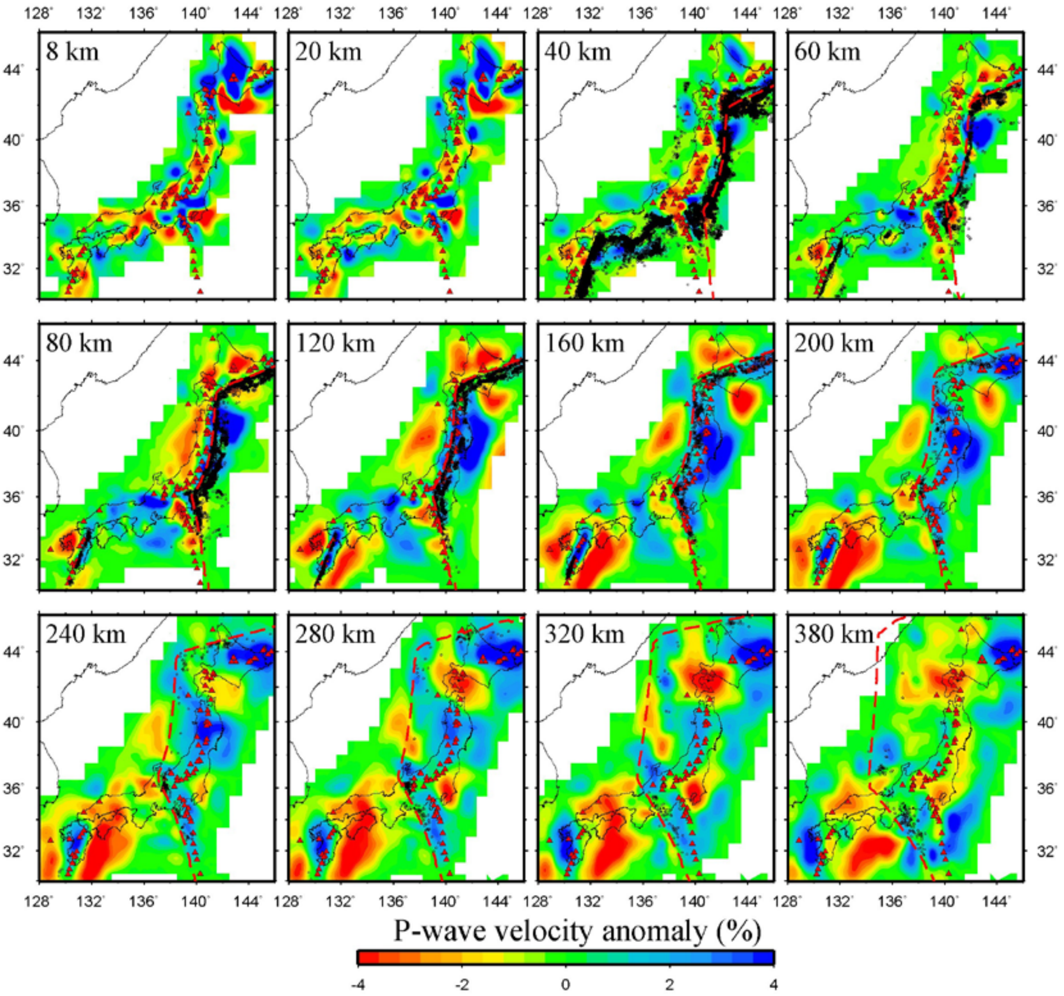

Figure S5. Map views of isotropic P-wave velocity tomography at different depths. The layer depth is shown at the upper-left corner of each map. The red and blue colors denote low and high velocities, respectively. The velocity anomaly (in %) scale is shown at the bottom. The red dashed line in each map shows the location of the upper boundary of the subducting Pacific slab at each depth[37, 38]. The red triangles denote the active arc volcanoes. The black circles denote the seismicity during a period of 2002-2007 that occurred within a 10 km depth of each layer, except for the layers at 8 and 20 km depths. This figure is generated using GMT 4.5.3 (http://www.soest.hawaii.edu/gmt/) developed by Wessel and Smith[59].

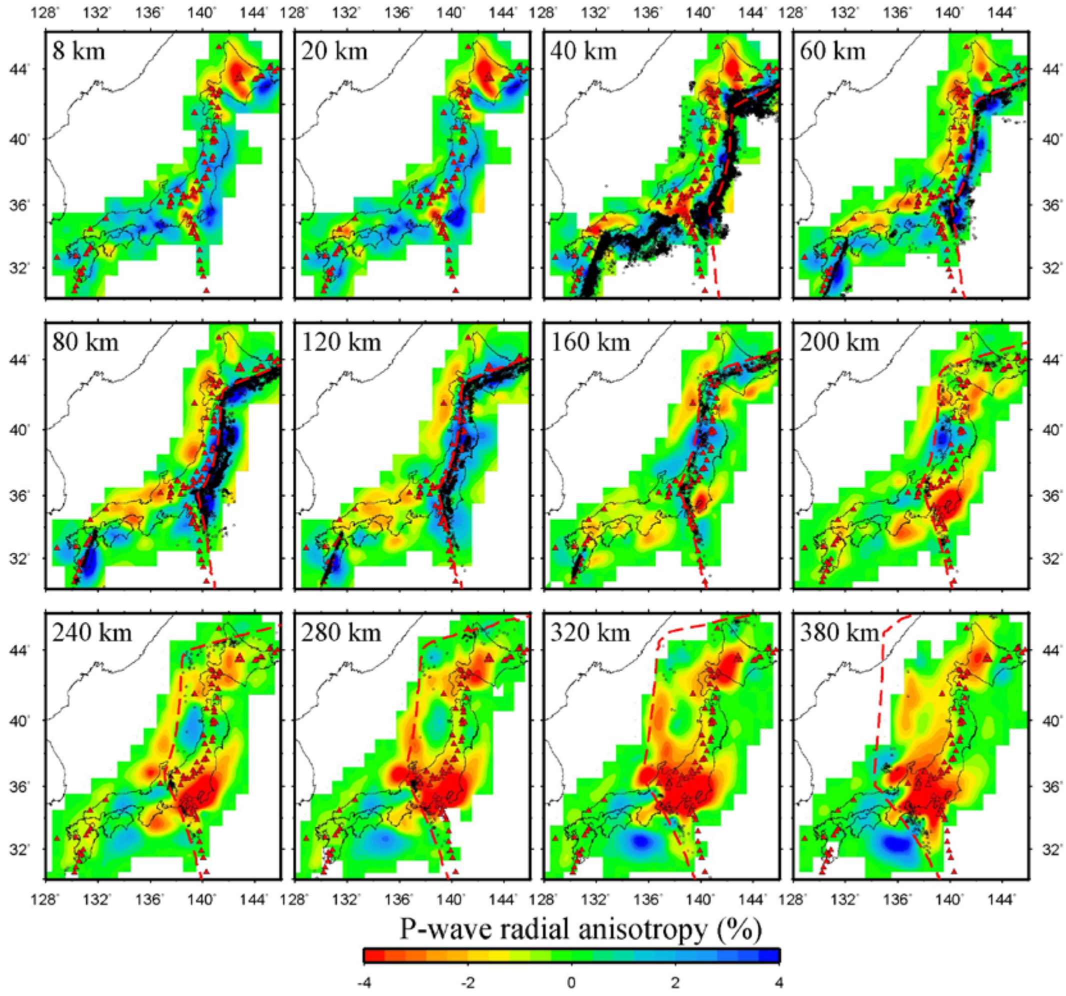

Figure S6. Map views of P-wave radial anisotropy at different depths. P-wave radial anisotropy is defined by $(V_{ph}-V_{pv})/(2V_{p0})$, where V_{p0} denotes the average isotropic velocity, V_{ph} and V_{pv} are velocities in the horizontal and vertical directions, respectively. The red and blue colors denote negative and positive radial anisotropies, respectively. Hence, negative radial anisotropy denotes P-wave traveling faster in the vertical direction, while positive radial anisotropy denotes P-wave traveling faster in the horizontal direction. The scale is shown at the bottom. The other labeling is the same as that in Figure S5. This figure is generated using GMT 4.5.3 (http://www.soest.hawaii.edu/gmt/) developed by Wessel and Smith[59].

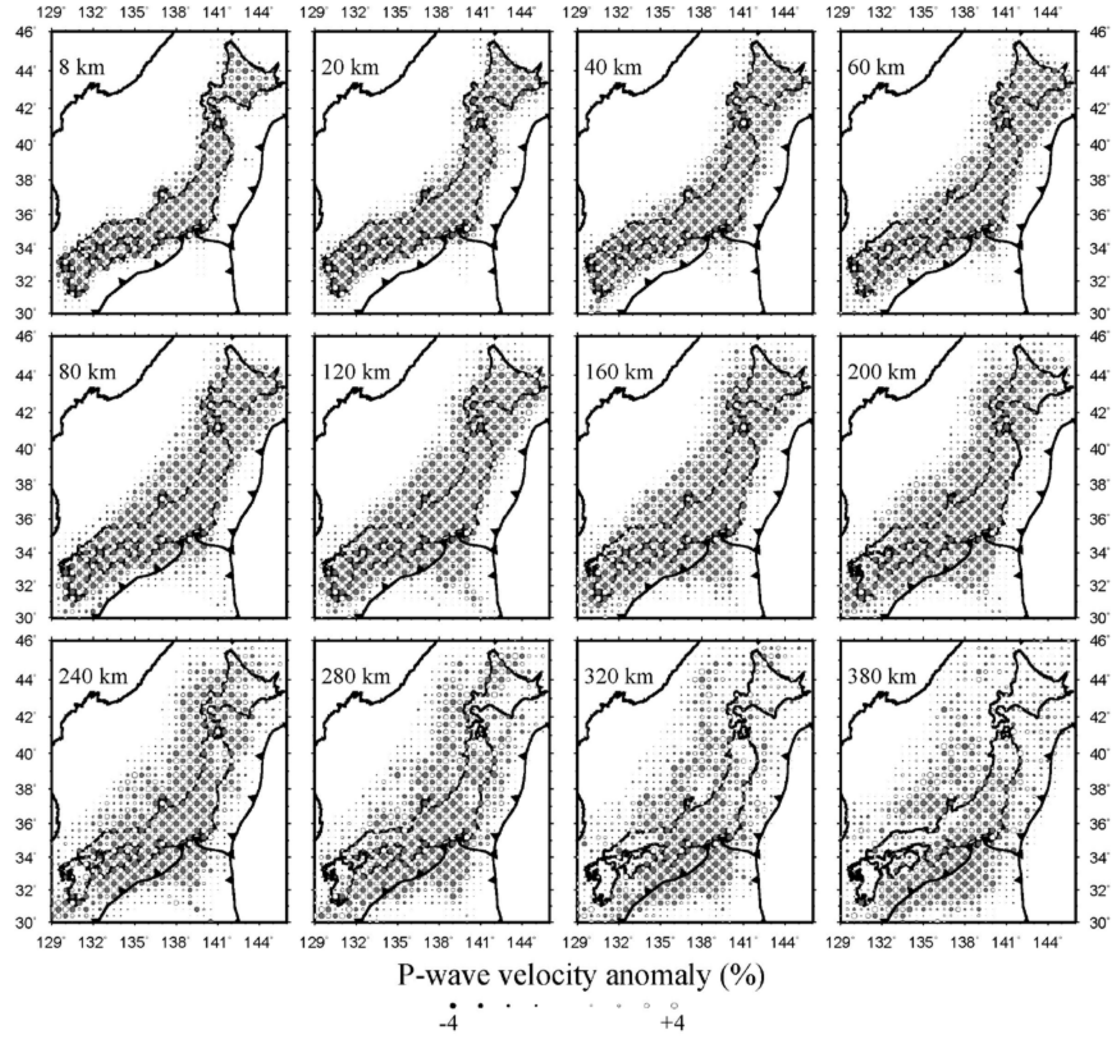

Figure S7. Results of the checkerboard test for the isotropic Vp tomography at different depths. In the input model, isotropic velocity anomalies of ± 4% are assigned alternatively to the grid nodes. The black and white circles denote low and high velocity perturbations, respectively. The scale is shown at the bottom. This figure is generated using GMT 4.5.3 (http://www.soest.hawaii.edu/gmt/) developed by Wessel and Smith[59].

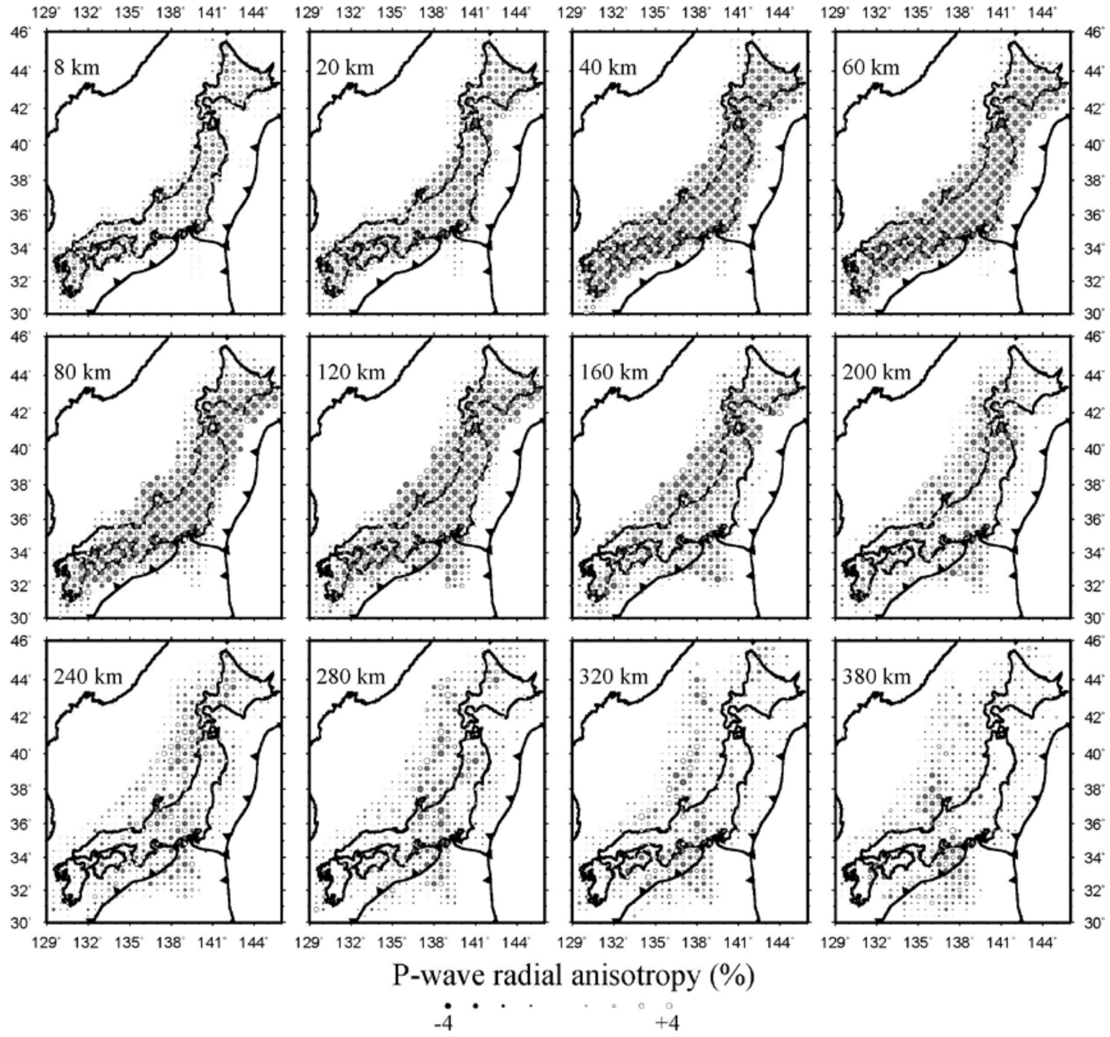

Figure S8. Results of the checkerboard test for P-wave radial anisotropy at different depths. In the input model, P-wave radial anisotropy of ± 4% are assigned alternatively to the grid nodes. The black and white circles denote negative and positive P-wave radial anisotropy, respectively. The scale is shown at the bottom. This figure is generated using GMT 4.5.3 (http://www.soest.hawaii.edu/gmt/) developed by Wessel and Smith[59].

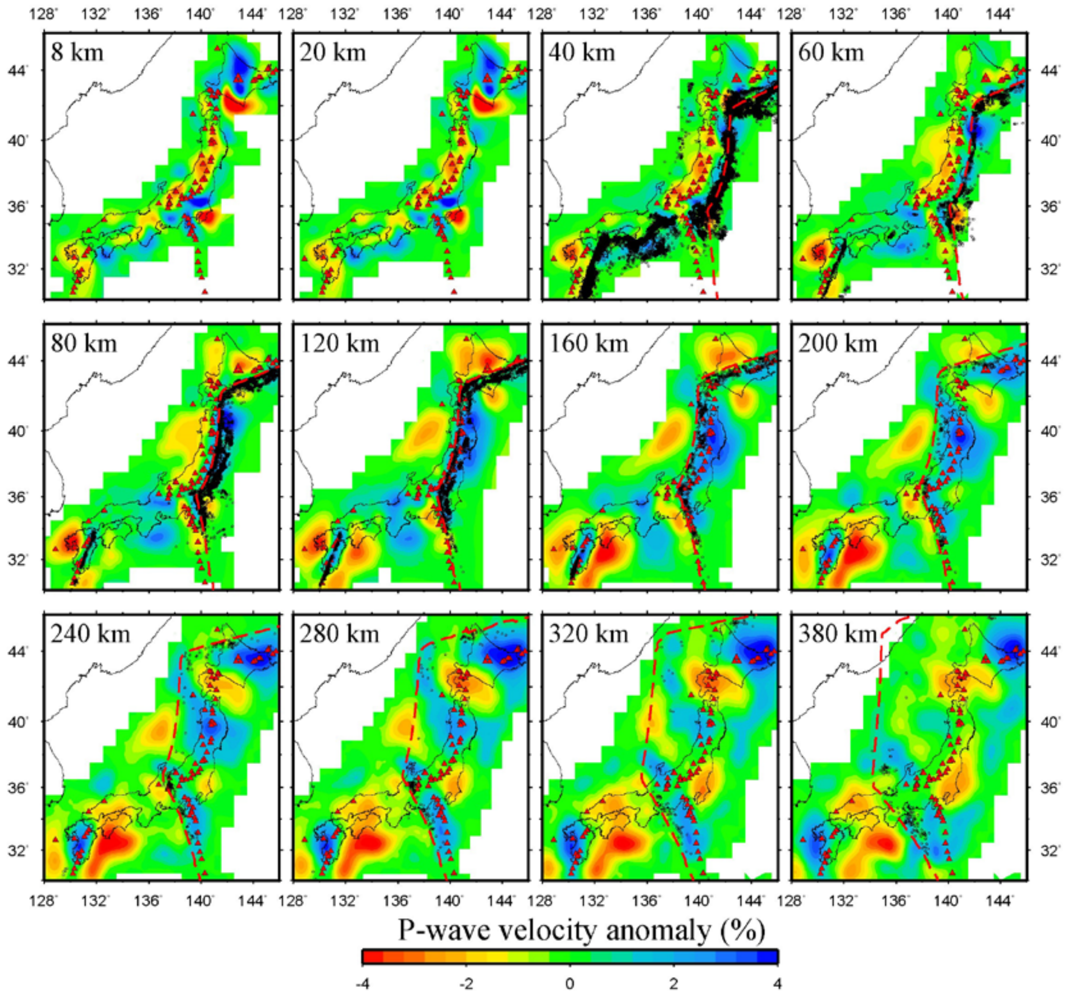

Figure S9. Results of the first synthetic test for the isotropic Vp tomography at different depths. In the first synthetic test, the obtained inversion results (shown in figures. S5 and S6) are adopted as the input model, and random errors in a normal distribution with a standard deviation of 0.15 s are added to the theoretical travel times to form a synthetic data set. The other labeling is the same as that in figure S5. This figure is generated using GMT 4.5.3 (http://www.soest.hawaii.edu/gmt/) developed by Wessel and Smith[59].

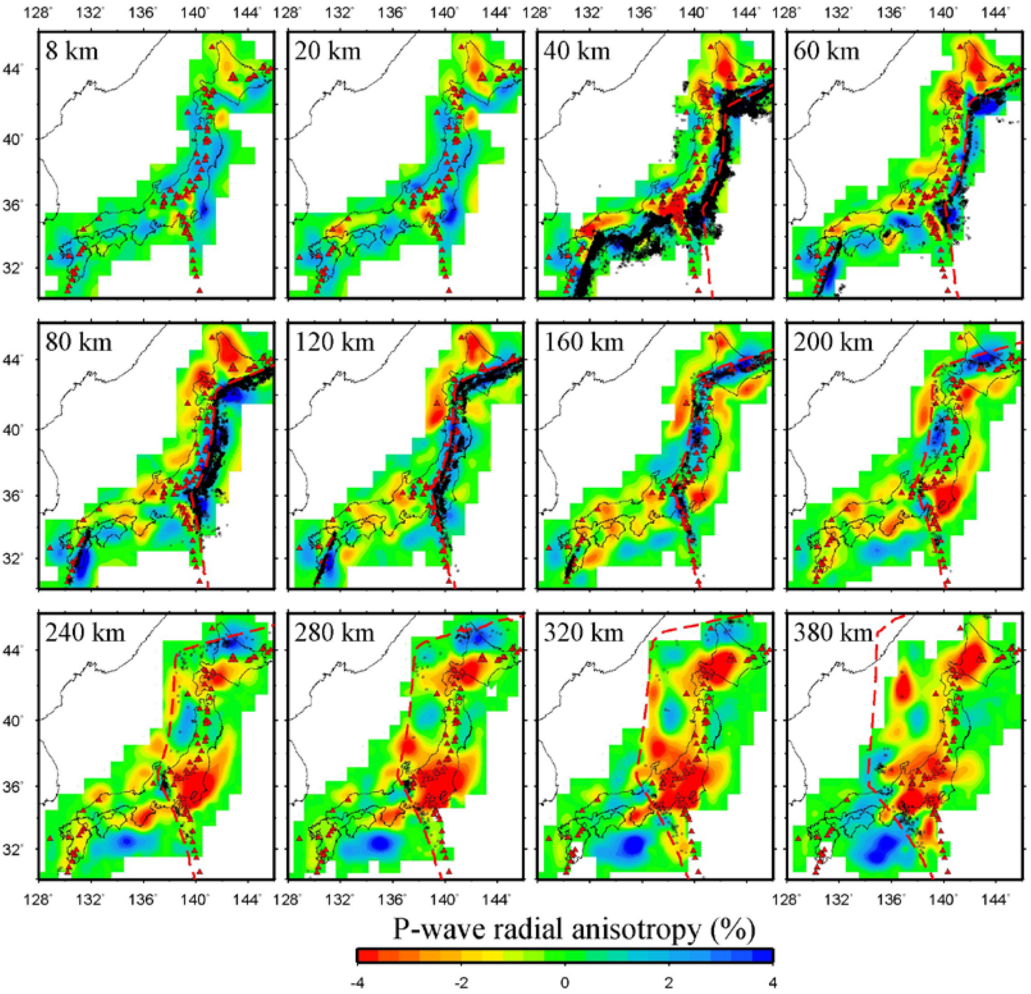

Figure S10. Results of the first synthetic test for P-wave radial anisotropy at different depths. The other labeling is the same as that in figure S6. This figure is generated using GMT 4.5.3 (http://www.soest.hawaii.edu/gmt/) developed by Wessel and Smith[59].

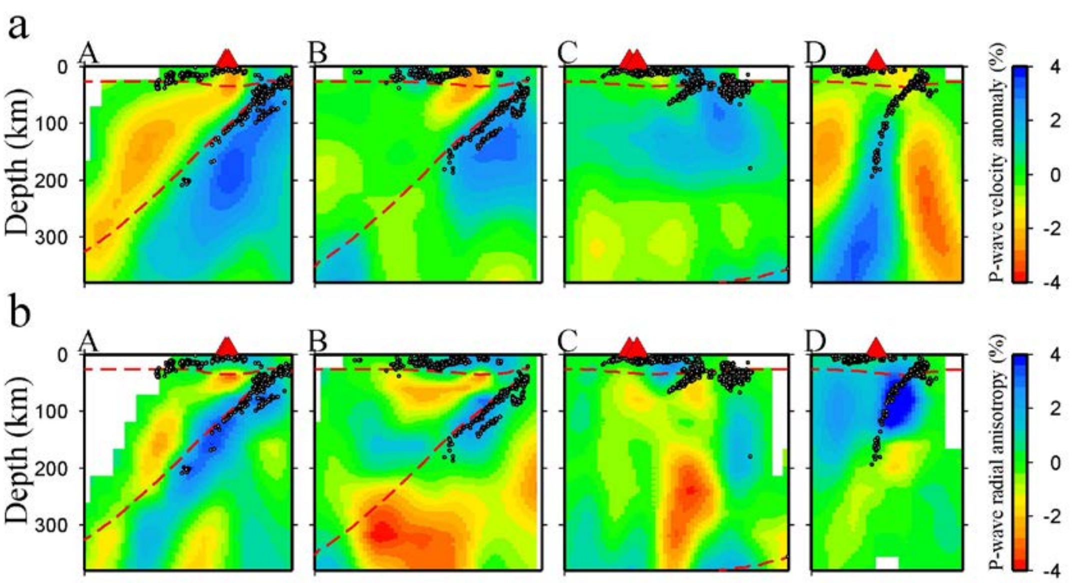

Figure S11. Results of the first synthetic test for P-wave velocity and radial anisotropy in vertical cross-sections. (a) Isotropic P-wave velocity images. (b) Images of P-wave radial anisotropy. The four profiles are shown in figure 1b. The definitions of the color scales for the isotropic velocity anomaly and radial anisotropy amplitude are the same as those in figures S5 and S6, respectively. The red dashed lines show the Moho discontinuity[33, 35] and the upper boundary of the subducting Pacific slab[37, 38], respectively. The red triangles and white dots denote active arc volcanoes and seismicity during a period of 2002-2007 within a 10 km width of each profile. This figure is generated using GMT 4.5.3 (http://www.soest.hawaii.edu/gmt/) developed by Wessel and Smith[59].

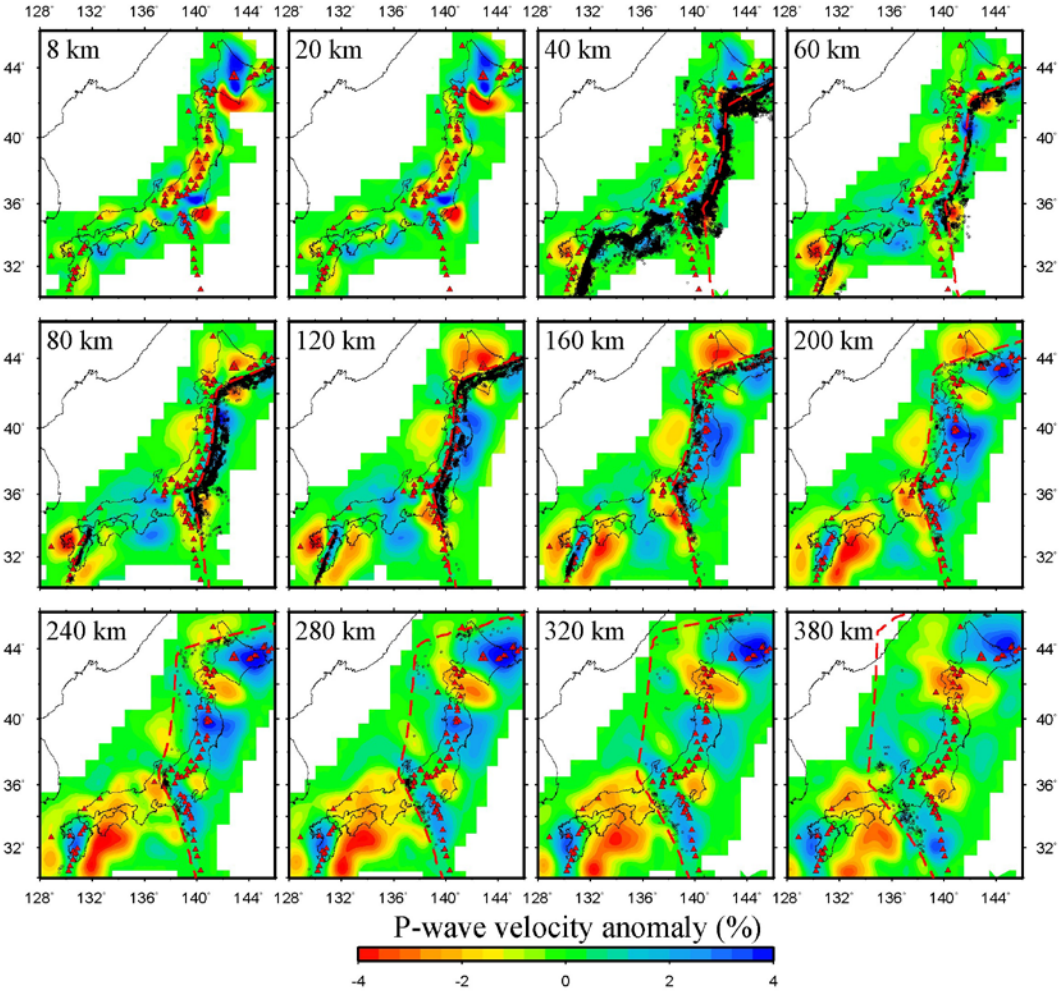

Figure S12. Results of the second synthetic test for the isotropic Vp tomography at different depths. In the input model of the second synthetic test, the velocity variations are the same as the obtained results shown in figure S4, but the radial anisotropy is opposite to the obtained results shown in figure S5. Random errors in a normal distribution with a standard deviation of 0.15 s are added to the theoretical travel times to form a synthetic data set. The other labeling is the same as that in figure S5. This figure is generated using GMT 4.5.3 (http://www.soest.hawaii.edu/gmt/) developed by Wessel and Smith[59].

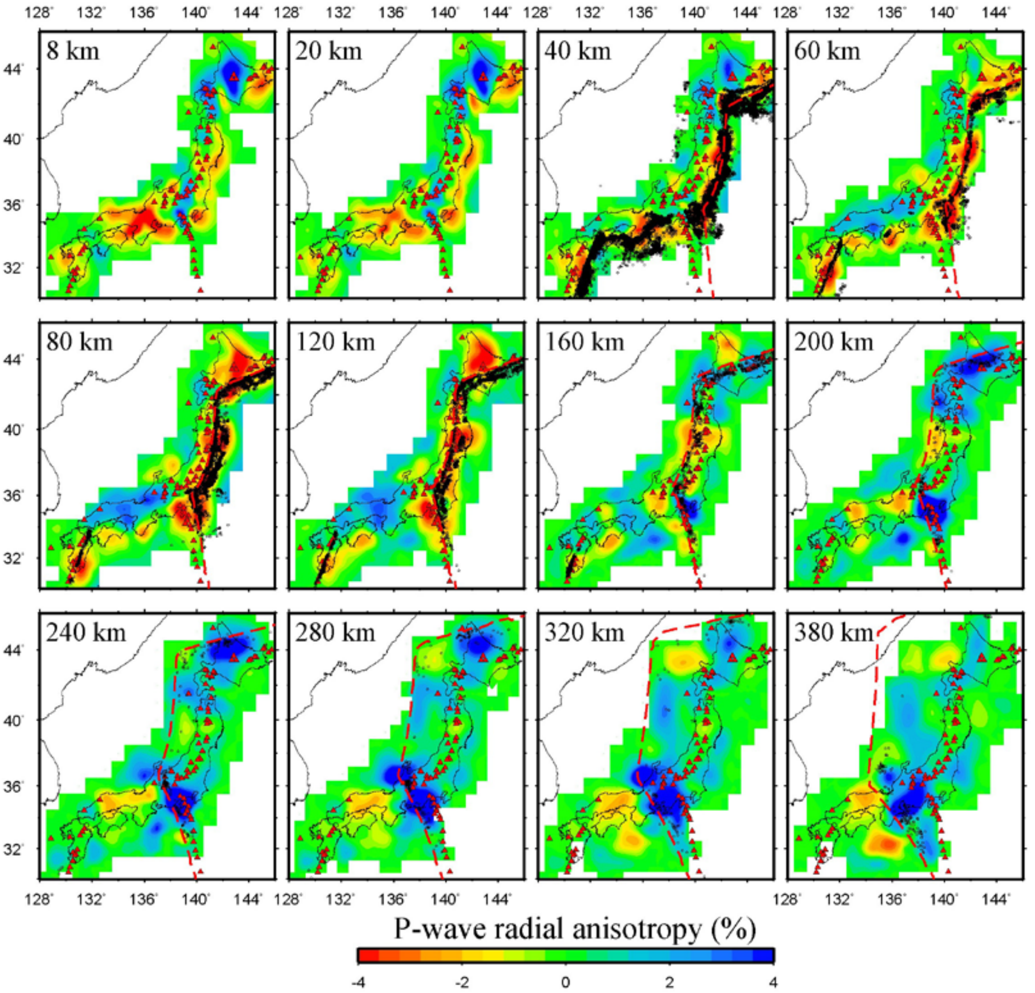

Figure S13. Results of the second synthetic test for P-wave radial anisotropy at different depths. The input model has a pattern of radial anisotropy opposite to that shown in figure S5. The other labeling is the same as that in figure S6. This figure is generated using GMT 4.5.3 (http://www.soest.hawaii.edu/gmt/) developed by Wessel and Smith[59].

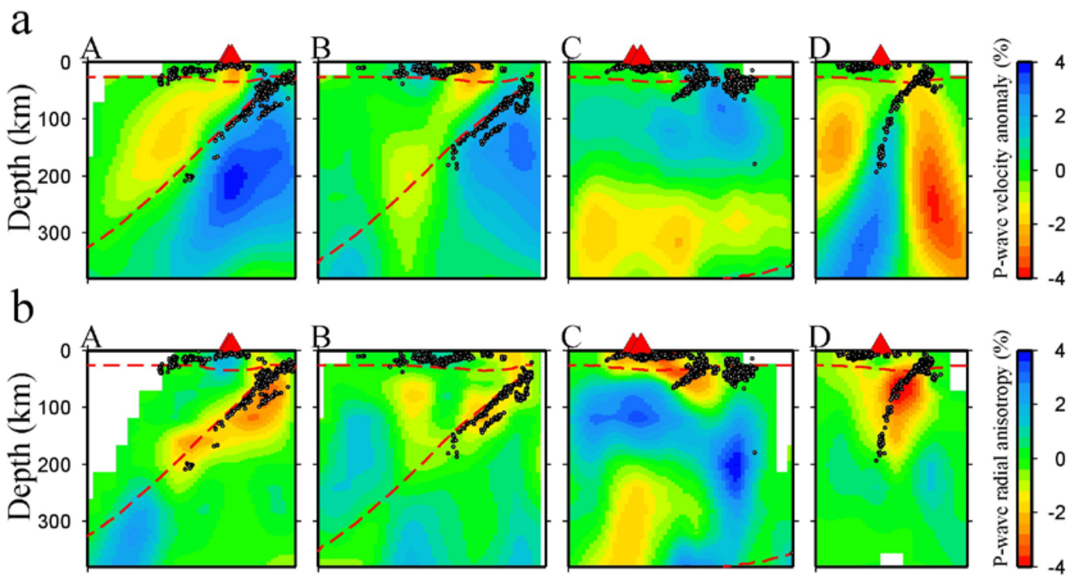

Figure S14. Results of the second synthetic test for P-wave velocity and radial anisotropy in vertical cross-sections. (a) Isotropic P-wave velocity images. (b) Images of P-wave radial anisotropy. The other labeling is the same as that in figure. S11. This figure is generated using GMT 4.5.3 (http://www.soest.hawaii.edu/gmt/) developed by Wessel and Smith[59].
